# Imaging Beta-Cell Function in the Pancreas of Non-Human Primates Using a Zinc-Sensitive MRI Contrast Agent

**DOI:** 10.3389/fendo.2021.641722

**Published:** 2021-05-26

**Authors:** Veronica Clavijo Jordan, Catherine D. G. Hines, Liza T. Gantert, Shubing Wang, Stacey Conarello, Christian Preihs, Sara Chirayil, Michael Klimas, Jeffrey L. Evelhoch, A. Dean Sherry

**Affiliations:** ^1^ Athinoula A. Martinos Center for Biomedical Imaging, Massachusetts General Hospital, Harvard Medical School, Charlestown, MA, United States; ^2^ Advanced Imaging Research Center, The University of Texas Southwestern Medical Center, Dallas, TX, United States; ^3^ Translational Biomarkers, Merck & Co., Inc., Kenilworth, NJ, United States; ^4^ Biometrics Research, Merck & Co., Inc., Kenilworth, NJ, United States; ^5^ Pharmacology, Merck & Co., Inc., Kenilworth, NJ, United States; ^6^ VitalQuan, LLC, Dallas, TX, United States; ^7^ Department of Radiology, The University of Texas Southwestern Medical Center, Dallas, TX, United States; ^8^ Department of Chemistry & Biochemistry, The University of Texas at Dallas, Richardson, TX, United States

**Keywords:** beta cell function, imaging, primate, diabetes, pancreas

## Abstract

Non-invasive beta cell function measurements may provide valuable information for improving diabetes diagnostics and disease management as the integrity and function of pancreatic beta cells have been found to be compromised in Type-1 and Type-2 diabetes. Currently, available diabetes assays either lack functional information or spatial identification of beta cells. In this work, we introduce a method to assess the function of beta cells in the non-human primate pancreas non-invasively with MRI using a Gd-based zinc(II) sensor as a contrast agent, Gd-CP027. Additionally, we highlight the role of zinc(II) ions in the paracrine signaling of the endocrine pancreas *via* serological measurements of insulin and c-peptide. Non-human primates underwent MRI exams with simultaneous blood sampling during a Graded Glucose Infusion (GGI) with Gd-CP027 or with a non-zinc(II) sensitive contrast agent, gadofosveset. Contrast enhancement of the pancreas resulting from co-release of zinc(II) ion with insulin was observed focally when using the zinc(II)-specific agent, Gd-CP027, whereas little enhancement was detected when using gadofosveset. The contrast enhancement detected by Gd-CP027 increased in parallel with an increased dose of infused glucose. Serological measurements of C-peptide and insulin indicate that Gd-CP027, a high affinity zinc(II) contrast agent, potentiates their secretion only as a function of glucose stimulation. Taken in concert, this assay offers the possibility of detecting beta cell function *in vivo* non-invasively with MRI and underscores the role of zinc(II) in endocrine glucose metabolism.

## Introduction

Glucose metabolism is tightly controlled by a systemic feedback-loop mechanism comprised of communication between beta cells in the pancreas and insulin-sensitive tissues (1). The disturbance of glucose homeostasis as a result of immunologically-induced loss of pancreatic beta cells ultimately leads to symptoms of type 1 diabetes mellitus (T1DM). This disease usually presents itself in young individuals, and common treatment options include self-injected doses of long-acting and short-acting insulin derivatives, mechanical monitoring of glucose and insulin dosing *via* pumps, and pancreatic islet transplantation (2, 3). In contrast, Type 2 diabetes mellitus (T2DM), responsible for approximately 90% of all diabetes cases (4), represents a systemic disease characterized by hyperglycemia either in the context of insulin resistance in peripheral tissues or lack of insulin secretion from the pancreas (1, 5). The treatment paradigm for T2DM includes initial lifestyle changes to attempt to reduce insulin resistance often supplemented with oral metformin, a biguanide-class drug whose primary mechanism of action is to reduce hepatic glucose production while simultaneously increasing insulin uptake in insulin-sensitive tissues (6). Although effective at reducing hyperglycemia, metformin has not been proven to prevent the conversion from impaired glucose tolerance to frank T2DM, only slowing the progression of disease while maintaining the rate of beta cell function deterioration when compared to other treatment options such as sulfonylureas or insulin injections (7). The pathophysiology of the disease is vastly heterogeneous across patients and this creates uncertainties in the optimal treatment regimen (1). While therapeutic outcomes may be predicted by knowing the state of beta-cell functionality (8), at this time there is no accurate way to measure beta-cell function directly from the pancreas in a non-invasive fashion.

Beta cell function is currently assessed by measuring blood glucose, proinsulin, insulin and C-peptide levels, or clamping experiments (9–12). However, blood levels lack information on the volume of beta cells (beta cell mass) or recruitment of cells (beta cell dynamics) needed to maintain blood glucose levels. While beta cell mass loss is well-known to parallel pancreatic function (13), it does not provide a complete picture of beta cell failure that is also attributable to beta cell dysfunction (14). Spatial or dynamic beta cell function information could be helpful to investigate the relationship of beta cell function to mass loss, where the latter can be measured invasively (15, 16) or non-invasively (17, 18). Both beta cell mass and function deficits contribute to T2DM (13) although some data suggests that beta cell function is more relevant and elusive in this disease (13, 14), particularly when beta cells are present but not viable (19). Thus, the means to non-invasively and longitudinally measure beta-cell function would be highly advantageous to determine the time course of T2DM development and for monitoring interventions to preserve beta-cell function.

Magnetic Resonance Imaging (MRI) is routinely used in abdominal diagnoses, particularly those related to the structural integrity of the pancreas. This includes ductal anatomy, presence of cysts, or acute pancreatitis, and diagnosis of lesions (20). Standard extracellular contrast agents are routinely used in abdominal MRI exams for tumor identification and staging. However, most if not all, currently conducted pancreatic radiological exams offer no direct information about beta cell mass or function. Zinc(II) is known to be co-released with insulin from beta cells in response to an increase in blood glucose. This ion is then dispersed into the islet extracellular space where it is taken up by neighboring alpha cells serving as an inter-cellular messenger ion signaling for glucagon secretion (21). The secreted zinc(II) can then be used as an indirect indicator of insulin release (i.e., beta cell function) by use of a zinc(II) responsive contrast agent. A number of MRI zinc(II)-responsive agents have been reported (22–25) with designs largely based upon formation of a ternary complex with serum albumin in the presence of excess zinc(II). Upon co-release of zinc(II) and insulin from pancreatic beta-cells, these agents form a complex with the excess zinc(II) and subsequently bind with albumin to form a high-relaxivity complex that ultimately translates into hyperintense voxels in a T_1_-weighted MRI image. Studies in rodents have demonstrated that this allows functional mapping of the insulin profile *in vivo* in healthy and diabetic rodents by MRI (22, 26, 27). However, it is generally appreciated that the rodent pancreas is an amorphous organ dispersed along the abdomen and interlaced between the intestines, stomach, liver, and spleen so it is exceedingly difficult to identify the endocrine lobes both accurately and consistently to monitor beta-cell function or loss in a longitudinal manner as a consequence of diabetes. As a result, a larger animal model in which the pancreas is a solid organ with easily identifiable lobes is paramount to further evaluate the use of such agents for functional imaging of glucose-stimulated insulin secretion.

A technique for imaging beta-cell function directly from different regions of the pancreas or other tissues could be quite useful for islet transplantation studies. Identification of dysfunctional islets in the pancreas along with accurate sorting of xenograft islets would most certainly benefit from an imaging method designed to assess beta-cell function in order to maximize the chances of graft success. In the treatment of T2DM, it would be beneficial to obtain beta-cell functional information to understand the pathogenesis of T2DM, treatment response and ultimately aid in the selection of improved T2DM therapies. In this work, we describe the use of the previously reported zinc(II) sensor (Gd-CP027) (22, 28) to measure beta-cell function in healthy non-human primates. In order to evaluate the sensitivity and specificity of this assay, we perform a modified graded glucose infusion (GGI) paradigm to initiate zinc(II)/insulin co-release in the presence of Gd-CP027 versus a control agent (gadofosveset) with similar albumin binding behavior and relaxivity but lacking in sensitivity to zinc(II).

## Materials and Methods

### Animal Preparation

All procedures were performed in accordance with our institution’s IACUC guidelines at the AALAC-accredited facility where the animals were housed. Adult, healthy male and female (n = 2, each) rhesus macaques were used for this study; alternative large animal models (e.g. swine, canines) were not used because primates allow for easier translation to human studies. All four animals were imaged on two separate days, Study 1 and Study 2, which were approximately three weeks apart to allow for adequate recovery after anesthesia and blood sampling. Animals underwent a 16 hour fast prior to the day of the MRI exam, and water intake was restricted two hours prior to anesthesia to facilitate easier visualization of the pancreas.

The animals were fed a High Protein Monkey Diet 5045 and High Protein Monkey Diet Jumbo 5047 biscuits once a day, where daily intake was calculated and tailored to each animal as a function of age, weight, and body condition. The animals weighed 7.1 ± 1.1 kg (range, 6.3-9.7 kg) on both study days. To facilitate handling in preparation for MRI, animals were first anesthetized with 10 mg/kg ketamine hydrochloride (100 mg/mL) administered intramuscularly so intravenous catheters could be placed into the left and right saphenous veins and a cephalic vein for contrast agent, glucose infusion, and blood sampling, respectively. Animals were then administered 5 mg/kg of propofol intravenously *via* the cephalic vein to allow endotracheal intubation. Once placed on the scanning table, anesthesia was maintained using a mix of approximately 70:30 ratio of oxygen to isoflurane, which was then maintained between 1.5-3%. A temperature probe, pulse oximeter and end tidal CO_2_ monitor were connected for continuous monitoring. Body temperature was maintained by a dorsal K-module warm water recirculating blanket. Fluid maintenance (10 mL/kg/hr Lactated Ringer’s solution) was co-administered in conjunction with the GGI platform during the MRI exam, as described in main text.

Breath-hold imaging was also performed on the anesthetized animals during the dynamic 3D T1-weighted images only. Animals were pre-selected based on their ability to breath-hold during previous MRI exams. The gas lines to the ventilator were shut off when breath-holding was needed and was then opened five seconds before breath-holding needed to end. All breath-holds were less than 40 seconds, and at least three minutes were allowed for recovery between breath-holds, consistent with our animal protocol. Each animal was able to receive enough oxygen and remain physiologically stable while under anesthesia. SpO_2_ was monitored for any irregularities. SpO_2_ did not drop lower than 95, which would require longer recovery between breath-holds and adjustment of anesthesia, if necessary. Adjustments to the amount of air administered, ratio of oxygen to isoflurane, and percent of isoflurane were carried out as needed to maintain stable anesthesia and breath-holding under the supervision of a veterinarian.

### MRI Acquisition

All imaging was performed using a Siemens 3T Trio and a four-channel flex coil. After a series of localizer images to position the region of the abdomen where the T1-weighted images would be acquired, T2-weighted axial and coronal single-shot turbo spin echo images were acquired with respiratory triggering to identify the pancreas. Using these spin echo images, higher resolution, fat-saturated T2-weighted turbo spin echo images of the pancreas were acquired with respiratory triggering in preparation for the T1-weighted 3D gradient echo dynamic sequence. For dynamic imaging, an axial, breath-held 3D T_1_-weighted (“T1W”) sequence was acquired with the following parameters: FOV = 20 cm x 20 cm, slice thickness = 1.0 mm, TE/TR = 2.32/5.75 ms, 1 average, 30 slices, parallel acceleration factor = 2, flip-angle = 10˚, 192 x 192 matrix for a resolution of 1.0 mm x 1.0 mm x 1.0 mm, BW = 360 Hz/pixel, and a scan time of 25 seconds.

In Study 1, the animals were administered 50 mg/kg/hr Gd-CP027 intravenously. For comparison purposes, this amounts to ~2-3 fold less contrast agent that that typically administered as a single bolus (0.1 mmol/kg) in a clinical study. Lyophilized, research grade Gd-CP027 (VitalQuan, Dallas, TX) was reconstituted in 100 mM TRIS buffer, pH 7.40 (25.5 mg/mL) and filtered for sterility. In Study 2, gadofosveset trisodium was administered intravenously at 50 mg/kg/hr. Commercially available gadofosveset was diluted into 100 mM TRIS buffer, pH 7.40 (25.2 mg/mL) and filtered for sterility. The two imaging studies were performed at least three weeks apart.

Infusion of contrast agent was initiated after all anatomical images of the pancreas and an initial T1W image was acquired. Immediately after initiating the infusion, the first dynamic T1W, breath-held image was acquired (“-20 min”). The infusion was then continued for the entirety of the exam. After 20 minutes of waiting to ensure the contrast agent was well-distributed and in steady-state, dynamic T1-weighted images were acquired beginning at “0 min” over one hour (“60 min”) at four-minute intervals (17 total time points). The 20 min pre-circulation time was established by imaging the kidneys every five minutes after infusion until constant enhancement was seen in the renal pelvis (data not shown).

### Graded Glucose Infusion and Blood Sampling

**Figure 1:**
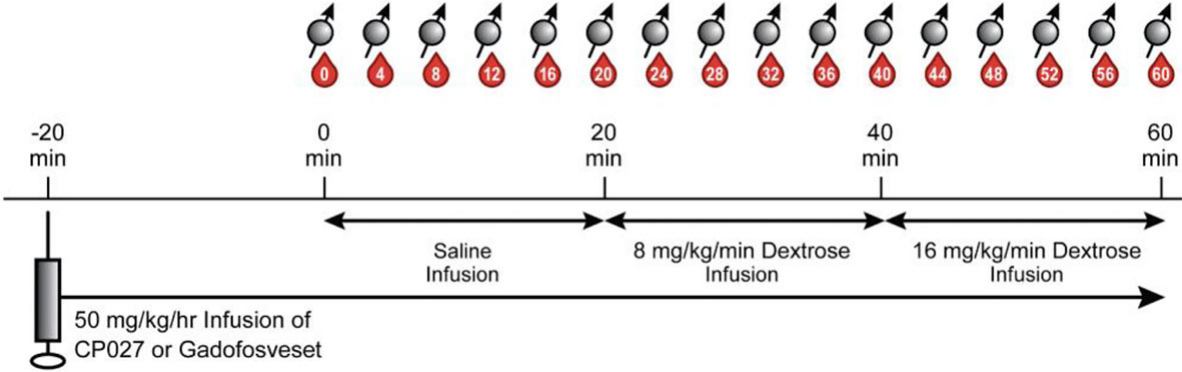
Altered GGI paradigm. After animals were anesthetized and anatomical images were acquired, 50 mg/kg/hr of contrast agent was infused intravenously. After waiting 20 min for the agent to circulate, a separate saline infusion was administered for 20 min (0 min – 20 min), followed by 20 min of 8 mg/kg/min dextrose (20 min – 40 min), and then 20 min of 16 mg/kg/min of dextrose (40 min – 60 min). Blood sampling (denoted as red drops) and breath-held T1W images (spin symbols) were acquired every 4 min.

To challenge the pancreas to co-release insulin and zinc(II), a modified 2-step graded glucose infusion (GGI) protocol, a standard paradigm for pancreas challenges (29–31), was performed during the MRI exam. Intermittent blood samples were also collected during the study for glucose, insulin, and C-peptide measurements. **Figure 1** summarizes the simultaneous MRI acquisition, blood sampling, and infusion paradigms. The GGI consisted of 20 min of saline infusion (0 min – 20 min) as an internal control, followed by 20 minutes of 8 mg/kg/min dextrose (20 min – 40 min), and then 20 minutes of 16 mg/kg/min dextrose (40 min – 60 min). Blood samples were taken every four minutes in EDTA tubes beginning at 0 min and continuing for one hour (60 min) at 4 min intervals.

### MRI Analysis

All images were analyzed using both VivoQuant (v2.50, inviCRO, LLC, Boston, MA) and ImageJ (National Institutes of Health, Bethesda, MD). The pancreas was manually subdivided into body/head/neck and tail regions of interest (ROIs), with the plane containing the superior pole of the left kidney identified as the division between the body/head/neck and tail. Small circular or ellipsoid-shaped hyperintense regions, typically 15-20 pixels in size, were observed predominantly in the pancreatic tail region during infusion of Gd-CP027 (n ≥ 4) and recorded. These regions are hereafter referred to as “hotspots”. During infusion of gadofosveset, a largely albumin-bound vascular agent, fewer and less intense hotspots were also detected but these likely reflect small vascular regions of increased blood flow stimulated by insulin secretion (32, 33). To compare the signal intensities of these hotspots with nearby regions of lower intensity, 4 – 7 similarly sized and shaped ROIs on the same imaging slice adjacent to the identified hotspots were also measured in images collected during infusion of either Gd-CP027 of gadofosveset. These ROIs are hereafter referred to as “within-slice comparator”). The signal intensity at each time point in all ROIs in the pancreas (whole pancreas, tail, body/head/neck, hotspots, within-slice comparators) was normalized to the signal intensity in nearby muscle ROIs at the same time points. Using the normalized signal intensities in the pancreas ROIs, the percent change from 0 min (initiation of saline infusion; hereafter referred to as “% Signal Change”) was calculated for each ROI at each time point.

Additionally, hotspots were categorized and clustered by size (area in mm^2^) using the “Grow region” tool on Horos dicom viewing software. The pancreas prior to contrast agent infusion was segmented and the average and standard deviation in signal intensity of a 5 mm-thick slab was measured. The smart ROI selection tool was programmed to only select regions of interest that were 2-standard deviations above the average segmented pre-infusion pancreas signal intensity. Every imaging time point was analyzed in this manner and the ROIs were identified by average signal intensity, standard deviation, and size. This sequence was repeated for all animals in both studies. A K-means clustering algorithm was performed in order to determine hotspot clusters by size and change in contrast-to-noise ratio as a function of blood c-peptide, where the centroid of the clusters was identified by minimizing the sum of the squared distance between the centroid and each data point.

### Blood Analysis

Plasma samples were analyzed for glucose, insulin, and C-peptide levels at each time point (In Vitro Sciences Laboratory, David H. Murdock Research Institute, Kannapolis, NC). Immediately after collection, blood was centrifuged at 1300 g at 4°C for 10 minutes. The plasma was removed, and frozen at -80°C for analysis. Glucose levels (mg/dL) were obtained using an Analox Glucose Analyzer (Analox Instruments Ltd, UK) from plasma samples and a commercially available glucose kit. Insulin levels (μIU/mL) and C-peptide levels (ng/mL) were obtained using a Meso Scale Discovery Sector Imager S 600 (Meso Scale Diagnostics, LLC, Rockville, MD) with commercially available insulin and C-peptide custom kits, respectively.

### Statistical Analysis

A sequential statistical analysis was performed using two models (34). The first model estimated the slope of each parameter vs. time for each contrast agent to characterize agent and time interaction (“slope analysis”) for C-peptide, glucose, insulin, and %signal change in the whole pancreas, head/body/neck, tail, hotspots, and comparator ROIs. Whenever the slope analysis identified differences between the two agents, a second analysis was performed. This second model used the means of each time point for each parameter and each agent to determine the time at which the two agents diverge (“time course analysis”). To avoid inflating false positives, rigorous multiplicity adjustments were performed and resulted in splitting the p-value between the slope analysis and the time course analysis. A p-value of 0.025 (=0.05/2) was used to determine statistical significance; a Bonferroni Correction for the multiple time point comparisons (n=15) in the time course analysis was applied using an adjusted significance level ~0.00167 (=0.05/2/15) to control the family-wise error rate at the 0.025 level.

## Results

**Figure 2:**
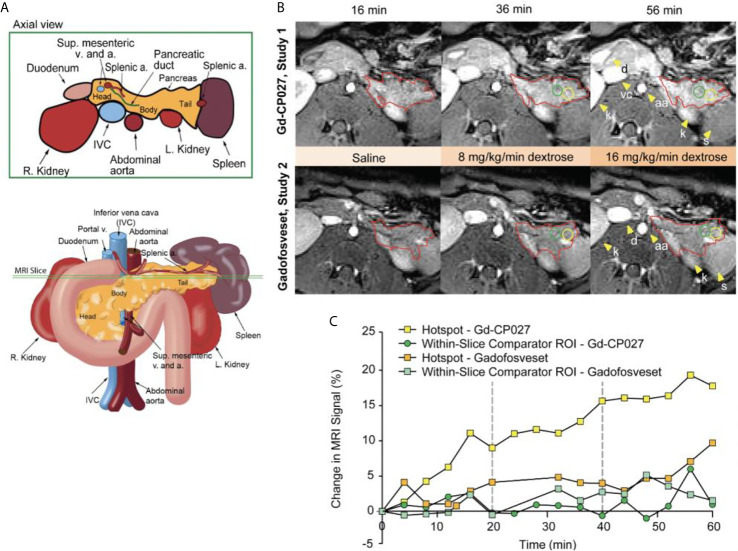
**(A)** Schematic illustrating non-human primate abdominal anatomy, including organs surrounding the pancreas, and an axial cross-section indicating imaging slice used for MRI pancreas localization and quantification. **(B)** In vivo MR imaging of non-human primate after infusion of Gd-CP027 (Study 1), and gadofosveset (Study 2). Both studies received the altered GGI, and 3D T_1_-weighted images were collected every four minutes. Axial images show the T_1_-weighted scans at 16, 36, and 56 min time points, where each represent the scan after complete saline infusion, 8 mg/kg/min dextrose, and 16 mg/kg/min dextrose infusion, respectively. The tail of the pancreas is highlighted in both panels (red) and Gd-CP027 shows a more prominent enhancement in the pancreas, with focal signal enhancements intensifying over time. Hotsposts (yellow) and adjacent comparator ROIs (green) were identified and measured, and are plotted in **(C)** for the animal displayed in **(B)**.

**Figure 2A** depicts the rhesus pancreas in vivo and plane of MRI acquisition shown in **Figure 2B**. **Figure 2B** shows representative T_1_-weighted MRI images acquired at the 16, 36, and 56-min time points corresponding to the end of the three different infusion blocks (i.e. saline, 8 mg/kg/min dextrose, and 16 mg/kg/min dextrose). In study 1, steady-state contrast enhancement was detected in the abdominal aorta and inferior vena cava by the end of the first infusion block (saline, 20 mins). Upon initiation of dextrose infusion at 8 mg/kg/min, the image intensity of the pancreas was further enhanced and small regions of higher intensity “hot spots” were detected primarily in the tail region. Signal enhancement throughout the pancreas and the hotspots became even more prominent when the dextrose level was increased to 16 mg/kg/min. The bottom panel of **Figure 2B** (study 2) shows images of the pancreas of the same animal and the same time points as in Study 1 after infusion of gadofosveset. Here, less enhancement was evident in images of the pancreas either before (16 min image) or after infusion of glucose (images at 36 and 56 min). **Figure 2C** compares the changes in image enhancement over the entire time course within the regions identified as hotspots after infusion of Gd-CP027 plus glucose versus gadofosveset plus glucose. All animals fully recovered from anesthesia after each study.

**Figure 3:**
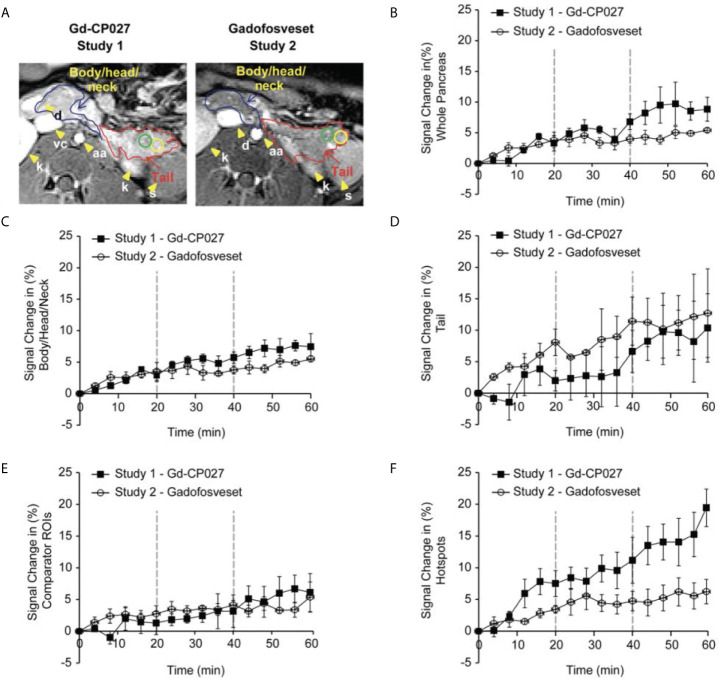
**(A)** Non-human pancreas segmentation delineating the tail (red outline), body/head/neck (blue outline) and ROIs within the tail. Change in signal intensity (%) after infusion of each agent alone (0-20 min) and after graded increases in glucose (20-40 and 40-60 min). for **(B)** the entire pancreas, **(C)** Body/Head/Neck, and **(D)** Tail of the pancreas. The error bars reflect standard deviations for the 4 animals. **(E)** Change in MRI signal intensity (%) for within-slice comparator ROIs for the 4 animals. **(F)** Change in MRI signal intensity (%) within the regions identified as hotspots in the 4 animals.

The average image intensity within ROIs consisting of the entire pancreas, the tail alone, and body/head/neck alone for the 4 animals are compared in **Figures 3A, B**. The increase in MR signal intensity over time in study 1 versus study 2 did not differ significantly for any of the segmented regions but an analysis of the slopes of these curves indicate there are statistically significant differences for the whole pancreas segment *versus* the body/head/neck region (p = 5.29E-7 and 2.79E-6, respectively). Like the comparator ROI data shown in **Figure 2C**, the segmented whole pancreas and body/head/neck time course data did not diverge significantly at any point during the GGI (p ≥0.00167 for all). The slope analysis for the tail region showed no differences between agents (p = 0.92).

**Figure 3** show plots of image intensity increase (± SD, n=4) for the segmented pancreas (**Figures 3B–D**) and for within-slice comparator ROIs (**Figure 3E**) and hotspot ROIs (**Figure 3F**) over the 60 min study. The hotspot ROIs showed a 3% increase in normalized signal intensity during infusion gadofosveset versus an 8% increase during infusion of Gd-CP027 over the first 16 min. These differences likely reflect differences in the extracellular distribution of gadofosveset (largely vascular) versus Gd-CP027 (both vascular and extracellular). However, after initiation of the GGI, these differences in ROI image intensity diverged even further with the gadofosveset data remaining near 5% at 60 min versus near 20% for Gd-CP027. These differences can only be attributed to responsiveness of Gd-CP027 to Zn(II) release in these well-defined regions of the pancreas. An analysis of slopes of these curves also reported significant differences [hotspot ROIs (p = 1.81E-12) versus comparator ROIs (p = 0.003)]. The hotspot data comparisons between the two studies diverged significantly at minute 44 (after infusion of glucose at the higher level) and remained divergent through the end of the GGI. The comparator ROI time courses did not diverge significantly at any time point during the GGI.

**Figure 4:**
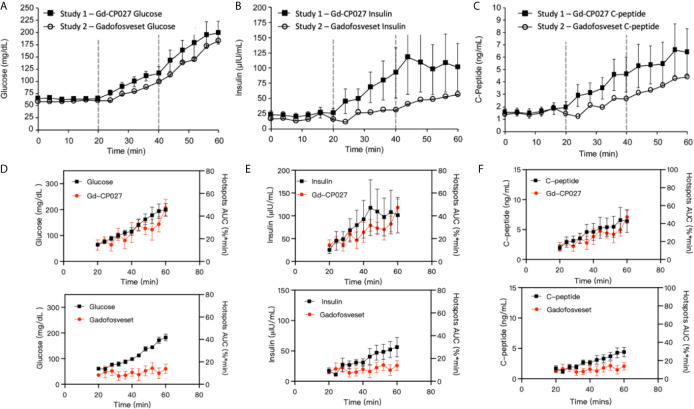
**(A)** Blood glucose, **(B)** insulin, and **(C)** C-peptide levels collected during each GGI study (n = 4). Error bars represent standard error, and vertical dashed lines reflect each infusion period. Area under the curve for Gd-CP027 and Gadofosveset hotspots overlapped to blood measurements of **(D)** Glucose, **(E)** Insulin, and **(F)** C-peptide.

The blood measurements showed the expected increase in blood glucose during each dextrose infusion period in both studies (**Figure 4A**). An analysis of slopes of these curves showed no significant differences between study groups (p = 0.07). As expected, insulin and C-peptide increased in parallel with plasma glucose (**Figures 4B, C**) although, interestingly, both insulin and C-peptide were consistently higher in the Gd-CP027 study after infusion of glucose in comparison to the gadofosveset study. The slopes of these time-dependent increases were significantly different for both insulin (p = 4.64E-5) and C-peptide (p = 3.29E-5). These differences indicate that Gd-CP027 enhances insulin secretion above that initiated by glucose. By analyzing the area under the curve of the hotspot signal for both Gd-CP027 and Gadofosveset we observe that only Gd-CP027 corelates tightly to the blood measurements, in particular to insulin and C-peptide (**Figures 4D–F**).

**Figure 5:**
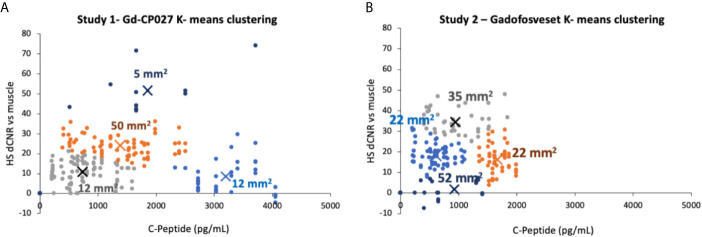
K-means clustering (k = 4 for both studies) showing the change in contrast-to-noise ratio versus the corresponding blood C-peptide measurement for all animals and all hotspots, where “X” represents the centroid of each cluster labeled with its respective average hotspot area for animals receiving **(A)** Gd-CP027, or **(B)** Gadofosveset.

To examine whether there are differences in the hotspot signal intensity and size patterns with respect to C-peptide levels in blood, plots of dCNR (increase in CNR compared to an equally-sized ROI in muscle) versus plasma C-peptide are shown in **Figures 5A, B**. Hotspots of various sizes were identified and included in this plot only if the average intensity was 2-standard deviations higher than the pre-infusion signal intensity. Further clustering of hotspot dCNR was done by using an algorithm to minimize the distance between the centroid of the clusters and each data point (K-means clustering). The same number of clusters (k = 4) was used in both plots. The average hotspot size in the Gd-CP027 group ranged from 12 - 50 mm^2^ in comparison to an average size of 22 - 52 mm^2^ in the gadofosveset group. One animal displayed hyper-physiological secretion of C-peptide/insulin in response to glucose as shown by the light blue colored cluster of 12 mm^2^ in sized hotspots in **Figure 5A**. This illustrates that not all animals release the same amount of C-peptide when stimulated by a similar increase in plasma glucose. Nonetheless, this K-means cluster analysis showed a positive correlation between hotspot dCNR versus blood C-peptide levels in the Gd-CP027 group but not in the gadofosveset group.

## Discussion

As demonstrated previously in isolated islets and in the rodent pancreas (26, 27), this imaging study of the non-human primate pancreas demonstrates that it may be feasible to assess β-cell function locally *in vivo* by glucose-stimulated zinc secretion (GSZS) and a Gd-based zinc(II) sensor as a contrast agent. Given that GSZS could also reflect β-cell mass, given the spatial resolution of MRI and the inability to resolve individual β-cells in this study it is deemed that this technology more accurately reflect β-cell function. Although MR signal enhancement was detected throughout the pancreas upon a graded glucose infusion (GGI) in the presence of either Gd-CP027 (zinc-sensitive agent) or gadofosveset (zinc insensitive), small regions previously referred to as “hotspots” (27) did show significant differences between the two agents during the highest GGI infusion period (44-60 min, **Figure 3F**). The blood plasma panel (**Figures 4A–C**) showed that glucose homeostasis was not significantly disturbed by infusion of either contrast agent (prior to initiation of the first glucose infusion) yet the insulin and C-peptide profiles suggest that Gd-CP027 may potentiate insulin secretion, especially at the highest level of glucose infusion. Since insulin secretion and potentiation by Gd-CP027 occurred only after infusion of glucose, the potentiation appears to be related to zinc(II) released in response to glucose and not the initial extracellular zinc(II) present in tissue. These observations are further confirmed in **Figures 4D–F** where the area under the curve (AUC) for the hotspots over the 20 – 60 minute infusion periods corelate tightly with insulin and c-peptide plasma profiles over time. However, further experiments will be necessary to understand the mechanism of Gd-CP027 potentiation, but it is important to note that neither Gd-CP027 nor gadofosveset are thought to enter cells so this potentiation likely reflects a disturbance in extracellular zinc(II) levels.

It is well known that zinc(II) plays a role in the correct function of many secretory organs including prostate, mammary glands, and the pancreas (21, 35–37). In the pancreas, co-secretion of zinc and insulin has been imaged using fluorescent probes (38, 39) and more recently by MRI (22, 24, 26). Given that MRI sensors detect insulin secretion indirectly by binding to excess Zn(II) released with insulin, it is important to consider the potential impact these agents have on other pancreatic secretory cell mechanisms. Secretory cells have distinct mechanisms to maintain tightly controlled Zn(II) homeostasis. This is mainly achieved by the expression of Zn(II) transporters of the ZnT and ZIP families (36). It has been shown that extracellular signals, such as exposure of prostate epithelial cells to an increase in plasma glucose (40), triggers a rapid redistribution of Zn(II) transporters between the plasma membrane and other cellular organelle membranes that results in also rapid redistribution of mobile Zn(II). Many other studies in multiple cell types have shown that alterations in various extracellular processes induces an immediate redistribution of intracellular Zn(II), a phenomenon referred to as “the zinc wave” (41). These and other studies showing that Zn(II) mimics the actions of hormones, growth factors, and cytokines have led to the concept that Zn(II) ions play a role as a second messenger capable of transducing extracellular stimuli into intracellular signaling pathways (42). Several different Zn(II) transporters are expressed in β-cells but the most highly abundant transporter, ZnT8, playing a key role in formation of zinc-insulin granules (43) and ZnT8 expression has been shown to positively correlate with circulating levels of insulin and glucagon (44). Although controversial, release of Zn(II) and insulin from β-cells has been reported to modulate glucagon secretion by uptake of Zn(II) ions into neighboring α-cells *via* ZIP transporters (21). Given that Gd-CP027 has a high affinity for Zn(II) (K_D_ ≈30 nM), infusion of this agent certainly must have an influence on the activity of Zn(II) transporters and likely could impact signaling of glucagon secretion (36, 45, 46). Although further studies will be required to determine the exact mechanism of insulin potentiation by Zn(II) chelating agents, we hypothesize that the origin of the observed potentiation of glucose-dependent insulin secretion by Gd-CP027 may reflect Zn(II) scavenging and subsequent calcium-mediated cell-membrane depolarization (21, 47). Although the plasma glucose levels tended to be higher in animals infused with Gd-CP027 versus gadofosveset (**Figure 4A**), these differences did not reach statistical significance. Nevertheless, this trend would be consistent with the hypothesis that a lower concentration of bioavailable Zn(II) in the extracellular space in animals exposed to Gd-CP027 could allow unabated glucagon secretion from α-cells.

One of the most interesting findings in this study was the focal nature of the normalized image enhancement in select regions of the pancreas. These focal hotspots, observed mostly in the tail, are consistent with clusters of islets as reported in previously published studies (27, 32, 33, 48). It is known that the regional distribution of pancreatic islets is uneven and clustered throughout the pancreas (32) and that this distribution varies among animal species (49, 50). The non-human primate, like the human pancreas, has insulin-secreting islets dispersed throughout the body of the pancreas, with higher β-cell density in the tail (50). Based on these prior reports, we expected to find significant differences in the segmented whole tail. An analysis of size and signal change clustering of hotspots (**Figure 5A**) showed that the smallest hotspots correlated with higher levels of C-peptide in blood even though there were no significant differences in image intensity in the entire segmented tail because of averaging with non-enhancing pancreas regions.

Although we observed that the change in signal intensity of hotspot clusters in the pancreas correlated with insulin and C-peptide secretion only in the Gd-CP027 group (**Figures 4D–F**), it was interesting to find observable hotspots in the pancreas in the gadofosveset group as well. These results indicate that these focal enhancements do not only reflect insulin and Zn(II) secretion but there must be an additional mechanism that contributes to these higher intensity regions in the gadofosveset study. Given that gadofosveset is a blood pool agent that extravasates into extracellular space more slowly than other low molecular weight Gd-based agents, it is reasonable to suggest that the focal enhancements observed in the gadofosveset study may reflect an increase in blood flow in those regions releasing the most insulin. It has been shown previously using fluorescence-labeled red blood cells that the apparent blood volume of the pancreas is greater during hyperglycemia than during hypoglycemia (51, 52) and this may be the origin of the effects observed here when using the vascular agent, gadofosveset.

The primary limitation of this study was the small number of animals available for study. Given the small cohort and the fact that one animal released more insulin and C-peptide while generating smaller changes in CNR in the Gd-CP027 study compared to the other animals, the differences between many of the parameters measured in this study did not reach statistical significance. Another potential limitation could be differences in pharmacokinetics and biodistribution of the two contrast agents. It was evident that uptake/clearance rates may differ, as seen in the 0-20 minute interval of **Figure 2C** and in the kidneys of **Figure 2B**, so the tissue biodistribution could contribute to local concentration differences at any given time point during the study. An analysis of slopes in the time-dependent data revealed significant differences between contrast agents for all measured parameters except the tail and glucose levels while the time course analysis revealed divergent responses at specific times only for the hotspot data. However, a consistent trend in the time course was evident in the plots (**Figures 3** and **4**) demonstrating different C-peptide and insulin release, as well as increased enhancement with Gd-CP027. This discrepancy between the sequential tests is due to a very conservative multiplicity adjustment as well as low power for the time course analysis due to the small sample size. Despite these limitations, this first study in the non-human primate pancreas offers the possibility of imaging β-cell function *in vivo* (38, 47, 53).

## Data Availability Statement

The original contributions presented in the study are included in the article/supplementary material. Further inquiries can be directed to the corresponding author.

## Ethics Statement

The animal study was reviewed and approved by the Institutional Animal Care and Use Committee at Merck & Co., Inc.

## Author Contributions

VCJ, AS, MK, JE, CH, and SCo conceived of the project. CH, LG, SW, VCJ, and JE carried out the experiments and analyzed the data. CP, SCh, and AS designed and prepared the zinc-sensitive imaging agent. All authors contributed to the article and approved the submitted version.

## Funding

This work was supported in part by grants to ADS from the National Institutes of Health (DK-095416) and the American Diabetes Association (7-12-IN-42).

## Conflict of Interest

CH, LG, SW, SC, MK, and JE were employed by Merck & Co., Inc. CP, one of the inventors of Gd-CP027, was employed by VitalQuan, LLC during the study. VitalQuan LLC licensed Gd-CP027 from the University of Texas and supplied the compound to Merck & Co., Inc. 

The remaining authors declare that the research was conducted in the absence of any commercial or financial relationships that could be construed as potential conflict of interest.
